# Relationship between Myopia Progression and School Entrance Age: A 2.5-Year Longitudinal Study

**DOI:** 10.1155/2021/7430576

**Published:** 2021-03-30

**Authors:** Linjie Liu, Dandan Jiang, Chunchun Li, Yaoyao Lin, Wenzhe Zhou, Haishao Xiao, Yanyan Chen

**Affiliations:** ^1^School of Optometry and Ophthalmology, Wenzhou Medical University, Wenzhou, Zhejiang, China; ^2^The Eye Hospital, Wenzhou Medical University, Wenzhou, Zhejiang, China

## Abstract

**Objective:**

To investigate the association between myopia progression and school entrance age among Chinese schoolchildren and to suggest a more appropriate school age.

**Methods:**

1,463 children aged six to nine years from Wenzhou, China, were examined and followed up for two and a half consecutive years. Their noncycloplegic refraction was measured twice each year by using an automatic refractometer; axial length (AL) and corneal radius of curvature (CRC) were tested annually by using the IOLMaster for 2.5 years. The questionnaires were completed by the children to collect detailed information regarding risk factors. Here, myopia is defined as a spherical equivalent less than −1.0D.

**Results:**

The changes in spherical equivalent (SE) of 7-year-old children in grade 1 and grade 2 were −0.45D and -0.56D, while changes in AL were 0.59 mm and 0.62 mm, respectively. The SE changes of 8-year-old children in grade 2 and grade 3 were −0.54D and −0.75D; meanwhile, the AL changes were 0.57 mm and 0.61 mm, respectively. Significant statistical differences were observed in ocular biological structure parameters, except for corneal radius of curvature (CRC) or anterior chamber depth (ACD), among children with the same age in different grades during this study. The prevalence of myopia was also significantly higher in higher grades for children with same age.

**Conclusions:**

Myopia is related to children's school entrance age. Children who start school in an earlier age are more likely to suffer from myopia, and the progression of myopia can be considerably faster. Therefore, it is recommended to enter school after the age of 7.

## 1. Introduction

In recent years, myopia has shown a high growth trend. Holden et al. [1] predict that, by 2050, the prevalence of myopia and high myopia will increase to 49.8% and 9.8%, respectively. Many studies [2–4] found that the myopia prevalence varies from country to country, among which the myopia morbidity rate in China is relatively high. Both genetic and environmental factors can play important roles in myopia development and progression, despite that their exact mechanisms are not yet known. Epidemiological studies [5–10] demonstrate that many environmental factors are associated with higher rates of myopia including not only ill habits of using eyes, larger amount of near work, or less outdoor activities but also academic burdens such as tests and schoolwork. Some scholars [11, 12] propose that early exposure to near work would consume children's hyperopia reserve and lead to early myopia, and Chinese children who live in a relatively competitive education system are often believed to be exposed to academic pressure earlier than the average level worldwide. However, no study was conducted on the relationship between school entrance age and myopia. Therefore, it is necessary to know the occurrence and progression of myopia in students of different grades and to explore the relationship between children's school age and myopia in order to investigate whether a proper school age can contribute to the control and prevention of the myopia among younger generations.

## 2. Methods

### 2.1. Study Settings and Participants

This prospective school-based investigational study chooses the longitudinal research design. Participants were followed up for 2.5 years, with the first visit in September 2012 to and the sixth visit in March 2015. The order by which each of the three schools was examined was the same from the first to the sixth visits to ensure a same gap between visits for each school. The detailed schedule of visits and examination items is shown in Supplemental Table 1. The cluster random sampling technique was adopted for recruiting participants. In the 2012 baseline measurements, 1523 students from grades one to three from three schools were included. Children with severe ocular diseases other than refractive error, such as congenital cataract, and children who would not cooperate with the examinations were excluded from the study.

The Wenzhou Epidemiology of Refraction Error (WERE) study was approved by the Institutional Review Board of the Eye Hospital of Wenzhou Medical University. Written informed consent was obtained from at least one parent or legal guardian of each participant.

### 2.2. Refraction and Ocular Examinations

Refractive error (noncycloplegic) was measured by using an autorefractor (RM-800; Topcon Corp., Tokyo, Japan) each semester. Ocular biological structure parameters including axial length (AL) and corneal curvature were measured with noncontact partial-coherence laser interferometry (IOL Master; Carl Zeiss Meditec, Oberkochen, Germany). Axial length (AL) was measured as the distance from the anterior corneal vertex to the retinal pigment epithelium along fixation, automatically adjusted for retinal thickness. Corneal radius of curvature (CRC), determined from the reflection of a hexagonal array of lights on the cornea, was measured along the flattest and steepest meridians.

All operators were well trained and assessed before conducting examinations. All examinations followed standard operating procedures and were supervised by the principal investigator.

### 2.3. Questionnaires on Children's Outdoor Activities and Near Work

To explore the causes for the changes in the patterns of myopia prevalence with school grade among students, we did a quick assessment in 2013 with questionnaire, taking a class as a unit. The questionnaires were distributed to students to record the times for their activities over the previous week. The collected demographic information included gender, grade, the child's lifestyle, and reading habits. The questions about lifestyles and reading habits pertained to near work activities and outdoor activity participation outside of school. Near work questions asked about the average time spent on near work each day, the distance from objects when doing near work, and whether the child had a 10 min rest after doing 30 min of near work, and questions about the average time of outdoor exposure after school on weekdays and on weekends was also included.

Questionnaire surveyors are trained uniformly, including the understanding of the questionnaire and the consistency of the explanation. The questionnaire will be collected by the investigator in the classroom, and the quality control and inspection will be carried out for each questionnaire to check and fill in the gaps to ensure the integrity of the data. All data of questionnaires were double entered to ensure the integrity and precision at the site.

### 2.4. Definitions

Spherical equivalent refraction (SER) is defined as spherical diopters (D) plus one-half of cylindrical diopters. In children aged 7–9 years, myopia is defined as SER ≤ −1.00 D. The reason of adopting the definition of myopia as a myopia refractive error of more than −1.00 D was that refractometry was carried out without cycloplegia; due to involuntary accommodation, the refractive measurements might have been artificially low in some children. The average corneal radius of curvature (CRC) was the average of the steepest and flattest meridians. The AL/CRC ratio was defined as the ration of the AL to the average CRC.

Cumulative shift in refractive error in each eye is determined by the difference in mean SER between baseline and follow-up measures (the follow-up measurement minus the original baseline measurement). Annual shift in refraction is the difference in mean SER divided by the mean follow-up time in years. Cumulative incidence of myopia is defined as the proportion of children who were not myopic (initial emmetropes and hyperopes) at baseline yet subsequently developed myopia during the follow-up period. The annual incidence rates were calculated by dividing the cumulative percentage by the mean follow-up time in years. Myopia progression is defined as a refractive change ≤ −0.5D/year.

### 2.5. Statistical Analysis

Data were analyzed by an SPSS software program (SPSS 23.0, Chicago, IL, USA). Since the refraction distributions of left eyes and right eyes were similar (Pearson coefficient = 0.864), only the data from left eyes were presented in this report. In both cohorts, SER, AL, and AL/CR ratios were compared by *t*-tests. The Pearson*χ*2 test was used to compare categorical variables such as near-work-related behaviors. Two-tailed *p* values were used in all analyses, and a *p* value of 0.05 or less was considered statistically significant.

## 3. Result

Inclusion of students in the baseline and follow-up:

1579 students from grade 1 to grade 3 (ages 6 to 10) were recruited from the three primary schools in Wenzhou. While students who would not cooperate with the examination and those who had severe eye diseases (*n* = 56) were excluded, a total of 1523 completed the baseline survey. In this study, 1080 children aged 7 years (538) and 8 years (542) at baseline were included in the analysis.

### 3.1. Change of Refractive Parameters and Ocular Components of Children of the Same Age in Different Grades for 2 Years

To present the 2-year change of refraction and refractive components, children were classified into four categories: grade 1 and 2 children at 7 years of age (Table 1); grade 2 and 3 children at 8 years of age (Table 2).

The mean SE of 7-year-old first- and second-grade children was 0.10D and −0.32D, −0.17D and −0.49D, and −0.30D and −0.71D for baseline, 1 year later, and 2 years later, respectively. Accordingly, the average AL was 22.74 mm and 22.95 mm, 23.01 mm and 23.25 mm, and 23.31 mm and 23.58 mm. Therefore, the AL/CRC ratios were 2.91 and 2.95, 2.95 and 2.99, and 2.99 and 3.03 at baseline, 1 year later, and 2 years later, respectively. Significant differences were observed in refractive parameters and ocular components excepting for CRC or ACD in students in grades 1 and 2 (Table 1).

The mean SE of 8-year-old second- and third-grade children was −0.29D and −0.52D, −0.40D and −0.73D, and −0.73D and −1.14D for baseline,1 year later, and 2 years later, respectively. Accordingly, the average AL was 23.21 mm and 23.39 mm, 23.47 mm and 23.67 mm, and 23.78 mm and 24.00 mm. Therefore, the AL/CR ratios were 2.96 and 2.99, 2.98 and 3.03, and 3.03 and 3.06 at baseline, 1 year later, and 2 years later, respectively. Significant differences were observed in refractive parameters and ocular components except for CRC or ACD in students in grades 2 and 3 (Table 2). However, Supplementary Table 2 shows there was no statistical difference in SE, AL, and AL/CR ratio among children of different ages in the same grade (*p* > 0.05).

### 3.2. Prevalence of Myopia of Students Aged 7-8 in Different Grades for 2 Years

**Figure 1:**
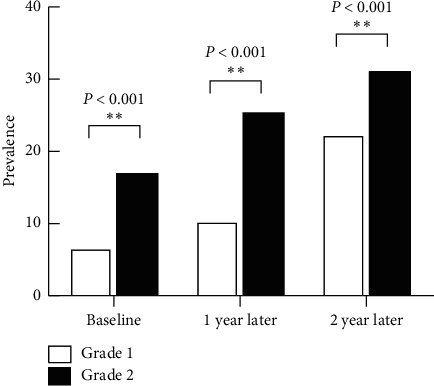
Prevalence of myopia among children of 7 years of age in grade 1 and grade 2.

**Figure 2:**
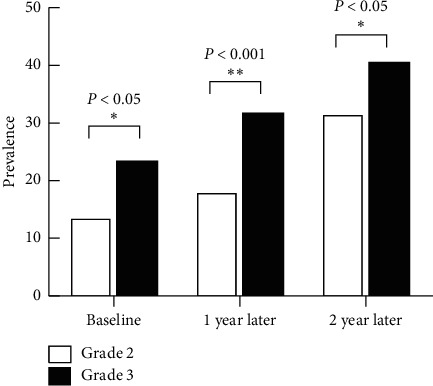
Prevalence of myopia among children of 8 years of age in grade 2 and grade 3.

As grade level increased, the prevalence of myopia also increased whether the students are 7 or 8 years old in baseline. The prevalence of myopia was also significantly higher in higher grades. The proportion of 7-year-old children who had myopia increased from 10.4% (56/538) to 22.3% (120/538), and the proportion of 8-year-old children increased from from 17.7% to 34.5% at the two-year follow-up. As is shown in Figure 1, the prevalence of myopia was 6.6%, 10.3%, and 22.3% in 7-year-old first-grade children and 17.00%, 25.4%, and 31.1% in second-grade children of the same age at baseline, 1 year later, and 2 years later, respectively. There was a significant difference between the different grades for the proportion of the prevalence of myopia. For the 8-year-old subgroup (Figure 2), the prevalence of myopia of grade 2 and grade 3 was 13.6% and 23.5% at baseline (*χ*2 = 8.734, *p* = 0.004); 1 year later, they became 18.1% and 31.8% (*χ*2 = 13.301, *p* < 0.001); 2 years later, they increased, respectively, by 31.6% and 40.6% (*χ*2 = 4.487, *p* < 0.05).

### 3.3. Myopia Progression and Change of Ocular Parameters of Children of the Same Age in Different Grades for 2.5 Years

In order to present the 2-year change of refraction and refractive components, children aged 7 and 8 years were classified into four categories according to gender and grade level (Table 3). The SE change of 7-year-old children in grade 1 and grade 2 was −0.45D and −0.56D, AL change was 0.59 mm and 0.62 mm, and the ratio of change AL/CRC was 0.07. For the change of CRC, a significant change was presented in children of 7 years of age in grade 1 and grade 2 as the grade level changed.

The SE change of 8-year-old children in grade 2 and grade 3 was −0.54D and −0.75D; the mean value of SE change in girls was −0.61D and −0.97D, and the value in boys was−0.49D  and −0.57D , respectively. AL change of 8-year-old children in grade 2 and grade 3 was 0.57 mm and 0.61 mm; among them, the AL change of girls was 0.59 mm and 0.67 mm; the average value of boys was 0.56 mm and 0.55 mm. There was a significant difference between the different grades for the change SE (*t* = 2.124, *p* = 0.034) (Table3). Furthermore, for an increase of a millimetre in AL, the amount of SE progression is larger for grade 3 (0.78D/mm) compared with grade 2 in the present population. With the increase of grade, SE and AL changed in female students, but no difference was found in boys. Except the change of CRC, significant differences in SE change, AL change, and AL/CRC ratio change were presented in girl student of grade 2 and grade 3.

### 3.4. Time Spent on Near Work and Outdoor Activities of Children of the Same Age in Different Grades

For the 7-year-old children, the average time spent on near work was 3.96 (SD = 1.67) h/day in grade 1 and 3.92 (SD = 1.46) h/day in grade 2. However, the average time spent on outdoor activities was 1.77 (SD = 0.86) h/day and 1.65 (SD = 0.80) h/day in 7-year-old grade 1 and grade 2 children. The average time spent on near work was 4.03 h/day and 4.14 h/day for 8-year-old children of grades 2 and 3. Also, the outdoor time was 1.75 h/day and 1.82 h/day for each grade. Time of near work increased steadily as grade level increased, but not statistically significant. On the contrary, time of outdoor did not change much with grade level (*p* = 0.78).

### 3.5. Time Spent on Continuing Various Kinds of Activities of Children of the Same Age in Different Grades

Table 4 displays the proportion of different grades in the same age with continuing to do various kinds of activities without an eye break less than 1 hour, 1 to 2 hours, and more than 3 hours. Significant differences in the proportion of time spent on continuous reading reading and writing was presented in children of grade 1 and grade 2 in the 7-year-old children (*χ*2 = 6.342, *p* = 0.042). There was also significant difference among 8-year-old children. Time of continuing playing computer, mobile phone games, and watching television, although constituting a quite large proportion of daily activity, did not change much with grade level. In addition, at the age of 8, there was a difference in the proportion of girls in grade 2 and grade 3 who watched TV continuously (84.6%, 10.8%, and 4.6% in grade two who were less than 1 h, 1-2 h, and more than 2 h, respectively; 78.4%, 20.6%, and 1.0% in grade three who were less than 1 h, 1-2 h, and greater than 2 h, respectively, *p* = 0.04), and the time of continuous watching of TV increased with grade level.

## 4. Discussion

Over the 2.5 years of this study, it was found that the prevalence of myopia was higher in higher grades among children of the same age, with smaller SE and longer AL. Axial length showed the strong correlation with SER, with longer eyes more likely to be myopic than shorter ones. It is also widely accepted that the age-related myopic shift in schoolchildren is mainly attributable to excessive axial elongation. Tideman et al. [13, 14] reported that AL predicts the onset of myopia, and the correlation between refractive errors was significantly stronger with the AL/CRC ratio than with AL and CRC alone [15–19]. Several studies [20–24] reported no significant change in the AL or AL/CRC ratio before and after pupillary dilatation. Therefore, the AL/CRC ratio can be a objective indicator of the onset and the progression of myopia [25]. Our results showed that compared with the lower grades subgroup, children in the higher grades subgroup had longer ALs and higher AL/CRC ratios. Also, children who were in higher grades of the same age are more likely to accelerate the occurrence and development of myopia.

The study discovered the children who started school earlier had longer time for continual and short-distance use of eyes, less reserves for farsightedness (hyperopia), and heavier academic burden. The association between advancing of school age and the occurrence and development of myopia may have the following two aspects.

The first is that it is the environmental exposures that a schoolchild receives, rather than their chronological age, that determine their refraction. Our study found that the number of words in textbooks and assignments that children in higher grades (about 800–900 thousand words/semester) need to learn is much higher than that in lower grades (about 600–700 thousand words/semester). With the increase of the grade, the course quantity gradually increased, with more heavy vision burden. Therefore, schoolchildren of the same age in different grades face different academic pressures. The association between educational stress and axial elongation can be quite strong. Also, children in higher grades had longer time for continual and short-distance use of eyes, suggesting that the time of continuing reading may be one of the main factors affecting the higher myopia diopter in higher-grade children. Our results also agreed with others [26–29]; as the grade increases, students continue to work closer together for longer periods of time, and the prevalence increases. Huang et al. [28] reported that continuing near work is one of the most critical influences for myopia progression. This may be explained by the fact that continuous near work may accelerate the occurrence and development of myopia.

Secondly, the children who enter school later have a higher tendency of hyperopia. With the increase of grade and the heaviness of academic pressure, the children of the same age who are earlier exposed to learning education, the cumulative effect is more. For example, compared to first-year students aged seven, second-year students aged seven had one more year of cumulative academic burden, including near work activities such as reading and writing. Meanwhile, some scholars [11, 12] propose that early exposure to close work would consume children's hyperopia reserve and lead to early myopia. The earlier the myopia occurs, the faster will be the progression of myopia and more likely it is to be highly myopic in adulthood. First of all, children of low age have poor hand fine movement ability. Most students use the pen improperly, read and write in the wrong posture, and it is difficult for them to learn to write Chinese characters horizontally and vertically [30, 31]. Secondly, since the development of children's nervous system and visual system is not perfect when they just enter primary school, the younger they are, the more likely they are to be affected by factors such as near work and study burden. Early exposure to near work may accelerate the occurrence and development of myopia. Furthermore, this study also found that compared with the lower grades, SE, AL, and AL/CR in female students who were in higher grades of the same age changed more over two years and the total time spent on near work increased, but there was no such difference among male students. The increase in near working hours makes them more likely to develop myopia [32]. With higher incidence and prevalence as reported in this study, female students appear to be at greater risk than male students. Similar findings have been reported by previous studies [33–37]; female students tend to read and write more and spend a greater amount of time indoors. Hence, female students constitute a high-risk group, and special efforts should be made to examine girls in this age group and also encourage them to play outdoors.

Early admission, the higher academic pressure, the heavier burden of close work, and the function and structure of the nervous system and visual system which are not fully developed lead to the students' sensitivity to the burden of doing intense close visual work, which increases the incidence of myopia and brings forward the onset age.

There are some possible limitations to the present study. Limitations of our study should be taken into account when its results are discussed. First, refractometry was not performed under cycloplegia, so that involuntary accommodation during refractometry might have covered a latent hyperopia. Myopia was defined as SER ≤ 1.00 *D* in our study to eliminate error. However, both AL and CRC can be measured accurately without cycloplegia. The fact that the higher AL/CRC ratios and higher prevalences of myopia appear in parallel suggests that cycloplegia is not a major problem. Second, the sample size of our study was convenient. Hence, further cohort studies with a larger sample size are warranted. Third, we need to follow this population into adulthood to see how the cumulative effect affects children of the same age in different grades. But, the current results have seen the trend that the children who start school in earlier age are more likely to suffer from myopia, and the progression of myopia can be considerably faster.

## 5. Conclusions

Myopia is related to children's school entrance age. Children who start school in an earlier age are more likely to suffer from myopia, and the progression of myopia can be considerably faster. Therefore, it is recommended to enter school after the age of 7.

## Figures and Tables

**Table 1:** Comparison of 2.5-year follow-up characteristics between 320 grade-one and 218 grade-two students who were 7 years old at baseline.

Characteristic	Baseline	1 year later	2 years later
SER (D)			
Grade 1: 7 years old	0.1 ± 0.8	−0.17 ± 0.84	−0.30 ± 1.25
Grade 2: 7 years old	−0.32 ± 1.3	−0.49 ± 1.62	−0.71 ± 1.75
T	4.139	2.655	2.88
*p* value^*∗*^	＜**0.001**	**0.008**	**0.004**

CRC (mm)			
Grade 1: 7 years old	7.8 ± 0.24	7.79 ± 0.23	7.81 ± 0.24
Grade 2: 7 years old	7.79 ± 0.26	7.78 ± 0.25	7.78 ± 0.26
t	0.678	0.31	0.265
*p* value^*∗*^	0.498	0.757	0.791

AL (mm)			
Grade 1: 7 years old	22.74 ± 0.74	23.01 ± 0.74	23.31 ± 0.83
Grade 2: 7 years old	22.95 ± 0.9	23.25 ± 0.99	23.58 ± 1.09
T	−3.015	−3.097	−2.993
*p* value^*∗*^	**0.002**	**0.002**	**0.003**

ACD (mm)			
Grade 1: 7 years old	3.26 ± 0.30	3.39 ± 0.25	3.41 ± 0.27
Grade 2: 7 years old	3.31 ± 0.27	3.41 ± 0.24	3.46 ± 0.25
T	−1.947	−1.439	−1.865
*p* value^*∗*^	0.052	0.151	0.063

AL/CRC			
Grade 1: 7 years old	2.91 ± 0.08	2.95 ± 0.08	2.99 ± 0.10
Grade 2: 7 years old	2.95 ± 0.10	2.99 ± 0.11	3.03 ± 0.12
t	−4.994	−4.386	−3.953
*p* value^*∗*^	＜**0.001**	＜**0.001**	＜**0.001**

SER, spherical equivalent refraction; CRC, corneal radius of curvature; AL, axial length; ACD, anterior chamber depth; *D*, diopters; values are means ± standard deviations. ^*∗*^The *t*-test was used to analyze the difference between grade groups.

**Table 2:** Comparison of 2.5-year follow-up characteristics between 332 grade-two and 217 grade-three students who were 8 years old at baseline.

Characteristic	Baseline	1 year later	2 year later
SER (D)			
Grade 2: 8 years old	−0.29 ± 0.93	−0.40 ± 1.19	−0.73 ± 1.45
Grade 3: 8 years old	−0.52 ± 1.29	−0.73 ± 1.37	−1.14 ± 1.81
T	2.256	2.655	2.757
*p* value^*∗*^	**0.025**	**0.008**	**0.006**

CRC (mm)			
Grade 2: 8 years old	7.86 ± 0.26	7.85 ± 0.25	7.85 ± 0.26
Grade 3: 8 years old	7.82 ± 0.24	7.82 ± 0.25	7.84 ± 0.26
t	1.614	0.31	0.489
*p* value^*∗*^	0.107	0.757	0.625

AL (mm)			
Grade 2: 8 years old	23.21 ± 0.83	23.47 ± 0.90	23.78 ± 0.96
Grade 3: 8 years old	23.39 ± 0.97	23.67 ± 1.02	24.00 ± 1.07
t	−2.217	−3.097	−2.461
*p* value^*∗*^	**0.027**	**0.002**	**0.014**

ACD (mm)			
Grade 2: 8 years old	3.33 ± 0.32	3.45 ± 0.25	3.48 ± 0.25
Grade 3: 8 years old	3.35 ± 0.29	3.49 ± 0.24	3.53 ± 0.26
t	−0.836	−1.439	−1.965
*p* value^*∗*^	0.403	0.151	0.05

AL/CRC			
Grade 2: 8 years old	2.96 ± 0.08	2.98 ± 0.09	3.03 ± 0.11
Grade 3: 8 years old	2.99 ± 0.10	3.03 ± 0.11	3.06 ± 0.12
t	−4.072	−4.386	−3.323
*p* value^*∗*^	＜**0.001**	＜**0.001**	**0.001**

SER, spherical equivalent refraction; CRC, corneal radius of curvature; AL, axial length; ACD, anterior chamber depth; *D*, diopters; values are means ± standard deviations. ^*∗*^The *t*-test was used to analyze the difference between grade groups.

**Table 3:** 2.5-year progression of myopia and changes in ocular biological structure parameters between different grades in the 7- and 8-year-old schoolchildren.

	Grade 1	Grade 2	Grade 3	t	*p* value^*∗*^
*Change SE, D*					
7 years old	−0.45 ± 0.99	−0.56 ± 1.22	N/A	1.038	0.3
8 years old	N/A	−0.54 ± 1.01	−0.75 ± 1.26	2.124	**0.034**

*Change AL, mm*					
7 years old	0.59 ± 0.34	0.62 ± 0.34	N/A	−1.822	0.069
8 years old	N/A	0.57 ± 0.37	0.61 ± 0.35	−1.108	0.268

*Change AL/Change CR*					
7 years old	0.07 ± 0.04	0.07 ± 0.05	N/A	−0.864	0.388
8 years old	N/A	0.07 ± 0.04	0.08 ± 0.06	−1.514	0.131

*Change SE/Change AL(D/mm)*					
7 years old	−0.38 ± 1.5	−0.33 ± 2.65	N/A	−0.253	0.8
8 years old	N/A	−0.25 ± 1.65	−0.78 ± 1.78	3.446	**0.001**

^*∗*^Comparisons of change of refraction and refractive components among different grades' students using the *t*-test.

**Table 4:** Comparison of variables among the grade groups in 7- and 8-year-old students.

Variables	7 years					8 years				
	Grade 1		Grade 2		*p* value^*∗*^	Grade 2		Grade 3		*p* value^*∗*^
	*n*	%	*n*	%		*n*	%	*n*	%	
*Continuous read-write time*										
<1 h	252	81.3	152	72.4	0.042	248	75.8	133	62.7	0.004
≥1 h and <2 h	34	11	38	18.1		58	17.7	62	29.2	
≥2 h	24	7.7	20	9.5		21	6.4	17	8	

*Continuous mobile game time*										
<1 h	277	89.6	182	86.3	0.303	266	81.8	182	85.8	0.419
≥1 h and <2 h	17	5.5	19	9		34	10.5	19	9	
≥2 h	15	4.9	10	4.7		25	7.7	11	5.2	

*Continuous use of computer time*										
<1 h	261	84.2	172	81.5	0.194	263	80.4	165	77.8	0.299
≥1 h and <2 h	27	8.7	28	13.3		34	10.4	31	14.6	
≥2 h	22	7.1	11	5.2		30	9.2	16	7.5	

*Keep watching TV*										
<1 h	263	85.1	177	83.9	0.091	250	76.7	157	74.4	0.259
≥1 h and <2 h	31	10	22	10.4		53	16.3	44	20.9	
≥2 h	15	4.9	12	5.7		23	7.1	10	4.7	

^*∗*^The Chi-squared test was used to assess the difference of variables between grade groups. *N* = number of cases, % = percentage of cases.

## Data Availability

The original data, figures, and tables that were used to support the findings of this study are available from the corresponding author upon request.

## Supplementary Materials

Supplementary Materials Supplementary Table 1 presents the timing of the examination visits. Supplementary Table 2 analyzes the baseline ocular biological structure parameters between different age students who were in the same grade at baseline. However, no more meaningful conclusions were reached.
