# Supplementary material for: Relationship between Myopia Progression and School Entrance Age: A 2.5-Year Longitudinal Study
Source: J Ophthalmol. 2021 Mar 30;2021:7430576. doi: 10.1155/2021/7430576 (PMC8026293; doi:10.1155/2021/7430576)
Supplement: Supplementary Materials — Supplementary Table 1 presents the timing of the examination visits. Supplementary Table 2 analyzes the baseline ocular biological structure parameters between different age students who were in the same grade at baseline. However, no more meaningful conclusions were reached. [file 7430576.f1.docx]

**Supplementary Materials**

Supplementary Table 1: The schedule of visits and examination items.

| Follow-up Time | Examination Items |
| --- | --- |
| Baseline  (September 2012) | Non-cycloplegic measurements of refraction (RM800); Ocular biological structure parameters including AL and CRC (IOL-Master) |
| 6 Months (March 2013) | Non-cycloplegic measurements of refraction (RM800) |
| 12 Months  (September 2013) | Non-cycloplegic measurements of refraction (RM800); Ocular biological structure parameters including AL and CRC (IOL-Master); Questionnaires |
| 18 Months (March 2014) | Non-cycloplegic measurements of refraction (RM800) |
| 24 Months  (September 2014) | Non-cycloplegic measurements of refraction (RM800); Ocular biological structure parameters including AL and CRC (IOL-Master) |
| 30 Months (March 2015) | Non-cycloplegic measurements of refraction (RM800) |

Supplementary Table 2: Comparison of Ocular biological structure parameters between different ages’ students who were the same grade at baseline

| characteristic | 6years old | 7years old | 8years old | 9years old | All | t | P value* |
| --- | --- | --- | --- | --- | --- | --- | --- |
| SER（D） |  |  |  |  |  |  |  |
| Grade1 | 0.14±0.81 | 0.09±0.92 | N/A | N/A | 0.06±0.87 | 0.613 | 0.54 |
| Grade2 | N/A | -0.32±1.30 | -0.29±0.93 | N/A | -0.30±1.09 | -0.295 | 0.769 |
| Grade3 | N/A | N/A | -0.52±1.29 | -0.42±1.49 | -0.47±1.40 | -0.770 | 0.442 |
| CRC（mm） |  |  |  |  |  |  |  |
| Grade1 | 7.81±0.24 | 7.80±0.24 | N/A | N/A | 7.79±0.25 | -0.717 | 0.474 |
| Grade2 | N/A | 7.79±0.26 | 7.86±0.26 | N/A | 7.83±0.26 | -3.020 | 0.003 |
| Grade3 | N/A | N/A | 7.82±0.24 | 7.82±0.26 | 7.82±0.26 | 0.201 | 0.841 |
| AL（mm） |  |  |  |  |  |  |  |
| Grade1 | 22.68±0.74 | 22.75±0.71 | N/A | N/A | 22.72±0.72 | -1.704 | 0.283 |
| Grade2 | N/A | 22.97±0.91 | 23.21±0.83 | N/A | 23.12±0.87 | -3.116 | 0.002 |
| Grade3 | N/A | N/A | 23.39±0.97 | 23.34±0.97 | 23.36±0.97 | 0.499 | 0.618 |
| ACD（mm） |  |  |  |  |  |  |  |
| Grade1 | 3.25±0.35 | 3.26±0.30 | N/A | N/A | 3.26±0.32 | -0.409 | 0.683 |
| Grade2 | N/A | 3.31±0.27 | 3.33±0.32 | N/A | 3.32±0.30 | -0.421 | 0.674 |
| Grade3 | N/A | N/A | 3.35±0.29 | 3.38±0.25 | 3.37±0.27 | -1.292 | 0.197 |
| AL/CR |  |  |  |  |  |  |  |
| Grade1 | 2.91±0.07 | 2.91±0.08 | N/A | N/A | 2.91±0.08 | 0.120 | 0.905 |
| Grade2 | N/A | 2.95±0.10 | 2.96±0.08 | N/A | 2.96±0.09 | -0.165 | 0.869 |
| Grade3 | N/A | N/A | 2.99±0.10 | 2.99±0.11 | 2.99±0.11 | 0.158 | 0.874 |
| SER, spherical equivalent refraction; CRC, corneal radius of curvature; AL, axial length; ACD, anterior chamber depth; D, diopters; values are means ± standard deviations. *t-test to analyze the difference between age groups. | | | | | | | |
